# The coherent and fluent mind: how unified consciousness is constructed from cross-modal inputs via integrated processing experiences

**DOI:** 10.3389/fpsyg.2015.00083

**Published:** 2015-02-09

**Authors:** Piotr Winkielman, Michał Ziembowicz, Andrzej Nowak

**Affiliations:** ^1^Psychology Department, University of California, San DiegoSan Diego, CA, USA; ^2^Behavioural Science Group, Warwick Business School, University of WarwickCoventry, UK; ^3^Department of Psychology, University of Social Sciences and HumanitiesWarsaw, Poland; ^4^Center for Complex Systems and New Technologies, Institute for Social Studies, University of WarsawWarsaw, Poland

**Keywords:** consciousness, coherence, fluency, subjective experience, cross-modal perception

Many philosophical approaches hypothesize that one function of consciousness is the creation of a unified subjective experience (Baars, 2005; Bayne, 2010). Such unified experience links different processing streams, originating in separate perceptual modules, thus enabling common access and generation of integrated decisions. All of this presumably occurs via a mechanism that blends information from different modalities into a single, multidimensional representation. But what exactly is unified in conscious experience? Prevailing explanations focus on integration of specific stimulus features at a perceptual or decisional level. In this opinion piece we discuss a simple but underappreciated explanation that focuses on processing dynamics. Specifically, we propose that cross-modal integration is facilitated by different modalities having a similar effect on the global subjective experience of processing quality. This integrated experience can then enter into decisional processes concerned with its source and relevance for the current behavior. As such, our account combines “experiential” and “decisional” process. Below we place this argument in the context of research on cross-modal integration and processing experiences, and discuss some implications.

Traditionally, research on multisensory experiences focuses on integration of information from different perceptual and conceptual cues. Some classic examples of such phenomena include the McGurk effect (changes in audition as function of vision; McGurk and MacDonald, 1976) and the double-flash illusion (changes in vision as a function of audition; e.g., Shams et al., 2000). Other classic examples of low-level cross-modal interactions include influences between pitch and brightness, loudness and size, or pitch and elevation. On a more conceptual level, cross-modal influences include shape or sound symbolism, such as the “bouba/kiki” effect (Ramachandran and Hubbard, 2001) and semantically-driven cases of synesthesia (Mroczko-Wąsowicz and Nikolic, 2014). A lively debate concerns when individual perceptual components from one modality are mandatorily modified by another modality, undergoing a low-level fusion that produces a single integrated percept, or when they are separate and integrated in high-level, post-perceptual stages via decisional processes (Spence, 2011; for an empirical example, see Hillis et al., 2002). Importantly, what such studies investigate are cross-modal influences on the representational content related to specific stimulus features.

Here we propose that cross-modal influences can also occur via processes that care less about the specific representational content but more about general representational quality, yielding global processing experiences. This proposal is grounded in several theoretical and empirical considerations.

Historically, the basic idea of processing experiences goes back to William James (1890) who spoke of “fringe consciousness” as experience that communicates a vague, unarticulated sense of peripheral contents relevant to the main task. Some “fringe experiences” include the feelings of familiarity and knowing, tip-of-the-tongue phenomena, and the sense of ease, rightness or coherence. Initially neglected by cognitive science, processing experiences and global “quality signals” are now of interest as a computationally efficient way of representing rich relational information (Mangan, 1993; Reber et al., 2002).

Empirically, the initial evidence for processing experiences came from research on fluency and familiarity (Whittlesea, 2002). For example, a pioneering study observed that people judge variable background noise as less loud when they hear a target word that was previously studied (Jacoby et al., 1988). Apparently, the ease (fluency) of target processing, deriving from previous exposure, gets misattributed to the loudness judgment. A related study reported misattributions of previous exposure to visual blur judgments (Whittlesea et al., 1990). Subsequent memory research documented similar influences using changes in perceptual format between stimuli appearing in the study and test phase of the experiment, including crossing words and pictures (e.g., Fazendeiro et al., 2005). Critically for the present argument, similar effects can occur for changes in modality, such as crossing auditory and visual stimulus presentation at study and test (e.g., Curran and Dien, 2003; Miller et al., 2008). These studies suggest that subjective experiences such as “fluency” and “familiarity” can be amodal and reflect joint influences from separate modalities. As a result, people cannot easily separate the processing quality associated with the target stimulus from contextual influences.

An important inspiration for our proposal are findings that a similar subjective experience can derive from processing facilitation at different processing stages. For example, factors that objectively facilitate visual detection and visual identification have similar effects on feelings of processing ease (Reber et al., 2004; Wurtz et al., 2008; but see Reber et al., 2014). As such, our proposal basically adds that experiential integration into a unified subjective feeling can occur even when the sources of processing experiences originate in different sensory modalities (e.g., quality signals from auditory processes can combine with quality signals from visual processes).

Importantly, our proposal assumes that experiential signals of processing quality can originate in processing of abstract, conceptual material, and extend beyond fluency (sense of ease) and familiarity (sense of oldness) to “structural experiences,” such as a sense of coherence, integrity, or rightness (Whittlesea, 2002). One example comes from research using artificial grammars and shows that decisions about grammaticality in one modality are influenced by previously learned grammatical rules in another modality, and that this influence involves non-analytical processes (Dienes et al., 2011). Importantly, this effect may not involve a feeling of fluency or familiarity, but rather a sense of structural coherence (Scott and Dienes, 2010). Stressing the breadth of such effects, our recent research shows that decisions about patterns in one modality can be influenced by the coherence of completely unrelated patterns from another modality (Ziembowicz et al., 2013). Let us elaborate as this research illustrates our core argument. In three experiments participants judged targets in one sensory modality while being incidentally exposed to regular or irregular background stimuli from a different modality. For example, targets were auditory melodies and backgrounds were visual figures, or vice versa. Critically, the specific regularity of targets and backgrounds was unrelated—auditory regularity was tone sequence grammar, visual regularity was 3D realizability. We explored the effect of cross-modal coherence with different types of subjective judgments: “regularity” (Experiment 1), “familiarity” (Experiment 2), and “possibility” (Experiment 3). All three experiments showed similar results: the coherence of the background stimulus influenced the target judgment, regardless of judgment type and target modality. That is, visual and auditory targets were judged as more “regular,” “familiar,” and “possible” when the incidental cross-modal backgrounds were coherent.

What are the implications of such findings? As mentioned, the standard explanation of cross-modal phenomena assumes changes in representation of stimulus features, whether driven by perceptual processes or decisional processes that integrate cues from different modalities. In contrast, we argue that cross-modal influences also reflect integration at the level of processing experiences. We admit the need for direct evidence that the just discussed cross-modal studies (including Ziembowicz et al., 2013) involve changes in subjective experiences and that their integration is causally responsible for the obtained behavioral effects. However, there is good evidence that related phenomena do involve “experiences”–i.e., cognitive or affective feelings. First, participants in many (though not all) experiments actually report changes in the feeling of “ease,” “effort,” “familiarity” or “regularity” associated with processing (Schwarz, 2015). Second, various physiological measures pick up indicators of changes in experience, such as positivity associated with fluent processing (e.g., Winkielman et al., 2003, 2012). Third, many experiments show that “bleed-over” or “misattribution” effects vanish once a person is provided with an explanation targeting subjective experience, not unlike classic studies on misattribution and discounting of affect or arousal (e.g., Dutton and Aron, 1974). For example in the previously mentioned cross-format study of Fazendeiro et al. (2005), participants were asked to recognize (old/new) words and pictures, some of which appeared earlier as related cross-format stimuli (essentially serving as semantic primes). During this recognition task, background music was played, which for some participants was explained as influencing their “sense of familiarity.” In this condition, participants showed reduced false recognition judgments for the cross-format stimuli, presumably reflecting their discounting of familiarity experience. Additional evidence for the notion that participants consciously experience changes in processing quality comes from research on hidden semantic coherence and the intuitive basis of such judgments (Topolinski and Strack, 2009a,b). Interestingly, this work shows that participants cannot report and re-attribute changing levels of fluency (facilitation due to semantic coherence) but are only aware of affective (hedonic) consequences of changed fluency. This suggests that what specifically is “felt” about objective processing quality varies depending on the details of the task. Finally, the just mentioned studies again highlight that the integration at the level of subjective experience interacts with high-level decisional processes. That is, the exact impact of experience on stimulus judgments depends on the perceiver's beliefs about the sources and relevance of the experience for the task at hand (Schwarz, 2015).

Neuroscientifically, our “joint quality signal” explanation for cross-modal integration matches evidence for global conflict signals or global prediction error (e.g., Fernandez-Duque et al., 2000; Friston, 2010; Shackman et al., 2011; Botvinick and Braver, 2015). Computationally, our account fits with connectionist models using global signals of processing quality (Lewenstein and Nowak, 1989; Norman and O'Reilly, 2003; Cleeremans and Dienes, 2008). Critically, these signals are non-specific, with different sources of coherence, ease, or familiarity generating a similar signal. Further, these signals are free-floating—not tightly bound to the original representation, and thus transferable across contents. Still, the signals are useful. They highlight abstract correspondences across patterns (e.g., regularity). They also regulate the network's own behavior, terminating the recognition process (preventing pattern discovery) when coherence is low and letting recognition continue when coherence is high (Rychwalska et al., 2005). The specifics of the mechanisms can be illustrated using a model by Lewenstein and Nowak (1989). It is a Hopfield type neural network enhanced with a mechanism allowing the network to control its own processing dynamics. The controlling system is implemented as a feedback loop that draws on one of a set of parameters: coherence, volatility, signal strength, etc. Based on the momentary values of this “order parameter,” the system can distinguish between known and unknown stimuli, but also react differently to primed, prototypical, regular, coherent, and distorted material. This model applies well to behavioral data, as seen in simulations of the mere exposure effect, which involves changes in fluency (Drogosz and Nowak, 2006). Consistent with behavioral data, the network reproduces the asymmetrical effect of “mere-exposed” stimuli on non-analytic, implicit, fluency-dependent judgments (preferences, familiarity) and analytic, explicit memory judgments. That is, the network results show that implicit measures of recognition (using the dynamic order parameter) can be faster than explicit measures, recreating a paradoxical phenomenon of somehow “knowing” the valence or familiarity of a stimulus before actually recognizing it.

In sum, we propose that some cross-modal phenomena involve integration via common experiences, including fluency, familiarity, and coherence, grounded in global signals about network dynamics. As a result, even when the modal origins of such signals differ, individuals experience integrated feelings of processing quality. Such feelings can then enter meta-cognitive processes and inform fundamental cognitive and social judgments (Winkielman and Schooler, 2011; Schwarz, 2015). Future research may explore cross-modal influences on experience-based judgments (risk, frequency, truth, fame, beauty, etc.). It should also determine when such effects are pre- and post-decisional. One question in this regard concerns the level at which processing signals are combined. It could be pre-experiential (e.g., fluency signals could blend before any experience) or experiential (e.g., with one already blended, or two blendable feeling signals appearing in the experience). A related question is whether experiences from different sources are genuinely fused (i.e., their origin information is lost) or potentially separable. Research should also explore the specificity of experiences. That is, sometimes experiences act broadly, allowing for conflation of drastically different inputs such as physical arousal with familiarity (Goldinger and Hansen, 2005) or physical effort with retrieval difficulty (Stepper and Strack, 1993). But, individual processing experiences are also unique in subjective quality (e.g., feelings of coherence differ from familiarity or ease, not unlike different emotions). This should constrain possible experiential fusion (genuine blending) and judgmental misattributions (source errors).

In conclusion, it appears that the creation of a unified consciousness is facilitated by an experiential mechanism that combines signals of processing quality. This mechanism links diverse contents in the mind and allows people to experience the multi-modal world as integrated—though also sometimes as more (or less) unified than it actually is.

## Conflict of interest statement

The authors declare that the research was conducted in the absence of any commercial or financial relationships that could be construed as a potential conflict of interest.
